# Spatial organization of hydrophobic and charged residues affects protein thermal stability and binding affinity

**DOI:** 10.1038/s41598-022-16338-5

**Published:** 2022-07-15

**Authors:** Fausta Desantis, Mattia Miotto, Lorenzo Di Rienzo, Edoardo Milanetti, Giancarlo Ruocco

**Affiliations:** 1 Center for Life Nano and Neuro Science, Istituto Italiano di Tecnologia (IIT), Viale Regina Elena 291, 00161 Rome, Italy; 2 Department of Physics, Sapienza University of Rome, Piazzale Aldo Moro, 5, 00185 Rome, Italy; 3 The Open University Affiliated Research Centre at Istituto Italiano di Tecnologia, Via Morego, 30, 16163 Genoa, Italy

**Keywords:** Biophysics, Molecular biophysics, Computational biology and bioinformatics, Protein folding

## Abstract

What are the molecular determinants of protein–protein binding affinity and whether they are similar to those regulating fold stability are two major questions of molecular biology, whose answers bring important implications both from a theoretical and applicative point of view. Here, we analyze chemical and physical features on a large dataset of protein–protein complexes with reliable experimental binding affinity data and compare them with a set of monomeric proteins for which melting temperature data was available. In particular, we probed the spatial organization of protein (1) intramolecular and intermolecular interaction energies among residues, (2) amino acidic composition, and (3) their hydropathy features. Analyzing the interaction energies, we found that strong Coulombic interactions are preferentially associated with a high protein thermal stability, while strong intermolecular van der Waals energies correlate with stronger protein–protein binding affinity. Statistical analysis of amino acids abundances, exposed to the molecular surface and/or in interaction with the molecular partner, confirmed that hydrophobic residues present on the protein surfaces are preferentially located in the binding regions, while charged residues behave oppositely. Leveraging on the important role of van der Waals interface interactions in binding affinity, we focused on the molecular surfaces in the binding regions and evaluated their shape complementarity, decomposing the molecular patches in the 2D Zernike basis. For the first time, we quantified the correlation between local shape complementarity and binding affinity via the Zernike formalism. In addition, considering the solvent interactions via the residue hydropathy, we found that the hydrophobicity of the binding regions dictates their shape complementary as much as the correlation between van der Waals energy and binding affinity. In turn, these relationships pave the way to the fast and accurate prediction and design of optimal binding regions as the 2D Zernike formalism allows a rapid and superposition-free comparison between possible binding surfaces.

## Introduction

Interactions between biomolecules are at the basis of every cellular process, from DNA replication to protein degradation^1–3^. To ensure a proper molecular recognition/function, proteins need to have a stable fold^4,5^ and form stable complexes^6^.

In both the cases of protein folding and protein–protein interaction indeed, the spatial arrangements of residues side chains give rise to a complex network of non-bonded atom-atom interactions, whose characteristics are expected to influence structure stability. The question arises of whether the structural and energetic properties of protein folding and binding are interrelated^7^. Indeed, the non-bonded (nb) intramolecular interactions in proteins, both in terms of their spatial and energetic arrangements, play a key role in the thermal stability of the protein structure^8–11^. Similarly, protein–protein intermolecular interactions are important for the binding affinity between the two interacting molecules^12–16^. To quantify the degree of folding and binding stability, two experimental descriptors are usually adopted: (1) the thermal resistance of each protein is typically evaluated through the melting temperature ($T_{m}$)^8^, while (2) the affinity of the interaction between two proteins is described by means of the equilibrium dissociation constant ($K_{d}$)^14^, often considered in logarithmic scale, i.e $B_{a}= log_{10}(Kd)$.

At the theoretical/computational level, instead, the prediction of the stability of a protein, even knowing its 3D structure, is still largely an open challenge since a complete understanding of the relationship between thermal resistance and the reorganization of the internal energies of the protein is still lacking^8^. In this context, some recent studies reported differences in terms of amino acid composition or spatial arrangement of residues, characterizing pairs of homologous proteins belonging to thermophylic and mesophylic organisms^17,18^.

In this respect, it must be noted that while proteins belonging to thermophilic organisms must have a $T_{m}$ higher than the optimal growth temperature of the organism, protein of mesophilic organisms can in principle have a much higher melting temperature than the one in which the organism thrives. Thus particular care should be used when performing such comparative analyses^9^.

Other studies showed that, even if core packing is related to thermal resistance^19^, the hydrophobicity of the residues plays a major effect in promoting/guiding the folding process while its contribution is smaller on protein stabilization^20,21^: the pivotal role for increasing the stability seems to be played by electrostatic residues on the protein surface^9^. Similarly, many computational methods have been developed to try to understand and, possibly, predict the basic mechanisms of the binding affinity between molecules^14,22,23^. For example, it was found that the presence of alanine residues anti-correlates with affinity, suggesting that such a residue will not provide favorable interactions^13^. On the contrary, the overall packing between two chains has been found similar to the packing observed within monomers^24^.

Studies on the role of salt bridges on the stabilization of binding proved that they can contribute significantly to the stabilization of some complexes while they produce destabilization of others^7^.

Altogether, the comprehension of the structural determinants of protein–protein binding is still far from being achieved. And this is also testified by the difficulties of predictive methods, principally those based on empirical functions typically used in molecular docking^25–27^, to successfully reproduce the results, especially when a large dataset is considered^28^. Therefore, the investigation of the energetic-structural properties that directly impact the binding affinity of a molecular complex plays a key role in understanding the nature of protein–protein interactions. Indeed, most of the effort in this field has been devoted to building energy functions for predictive purposes^12,29–31^. Moreover, many of these methods rely on a continuum framework and make use of molecular surfaces, the computation of which could affect the outcome depending on the used parameters^15^. On the other hand, to our knowledge, very few works have dealt with this problem under a statistical perspective or formalizing energetic properties in a set of descriptors^13,15^. To this end, here, we investigate the relationship between non-bonded intramolecular and intermolecular interactions that take place between the residue side chains to better elucidate their effect in structural stabilization^32^ of both protein folding and protein–protein binding^33^. In this scenario, the energetics of nonbonded atomic interactions can be summarized mainly by Coulomb and van der Waals forces.

To do so, we assembled two datasets of experimentally resolved molecular structures, the first composed of single-chain proteins for which the melting temperature value is reported ($T_{m}$ dataset)^9^ and the second one composed of protein–protein complexes of known experimental dissociation constant $K_{d}$ ($B_{a}$ dataset)^34^. We found that the more stable the monomeric structure the more it possesses favorable Coulomb interactions, while high favorable van der Waals interactions are associated to less stable proteins. A quite opposite behavior is present in relation to the binding affinity between two interacting molecules, where van der Waals’s contribution positively correlates with the complex stability. Moreover, an analysis of the amino acid composition of the binding sites confirmed a higher presence of hydrophobic residues and we quantitatively show that the higher the hydrophobicity of the residues the more van der Waals interactions account for the observed binding affinity. Finally, we focus on the role of van der Waals energy in the binding affinity, and on the connection between the van der Waals description of the molecular interfaces and the corresponding shape complementarity between the two interacting molecular surfaces.

In this framework, it is worth noting that several methods have been developed to measure geometric complementarity, ranging from the Katzir molecular surface^35^ to the Sc surface complementarity parameter^36^ or to the calculation of van der Waals intermolecular energies^37^. Here, we used the Zernike 2D method^38^, which can compactly and efficiently describe the geometric properties of portions of the molecular surface without the need of superimposing the two interacting protein structures.

## Results

### Intra- and intermolecular interaction energies in thermal stability and binding affinity

Firstly, we evaluated the Coulombic (C) and Lennard Jones (LJ) interaction energies between all couples of residues of the structures forming the $T_{m}$ dataset, i.e. all the non-bonded intramolecular interactions, and between all the residues of the $B_{a}$ dataset complexes, which will be referred to as intermolecular interactions. Details on how these energies were computed are reported in the “Methods” section.

**Figure 1:**
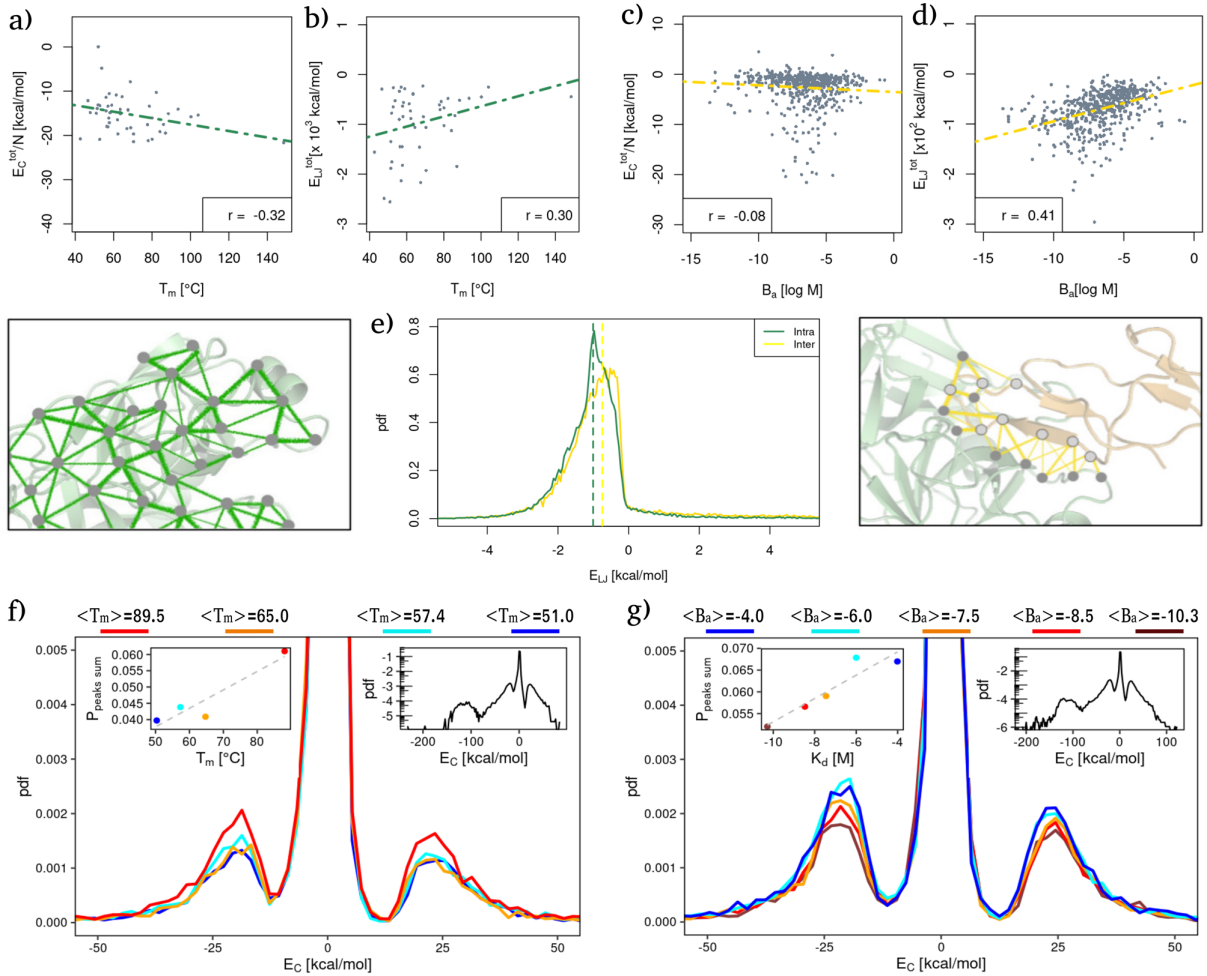
Comparison between intra and intermolecular interaction energies with respect to folding and binding stability. (**a**) Total Coulombic energy as a function of $T_{m}$ for each protein of the $T_{m}$ dataset. The Pearson correlation is reported in the legend. Energies are normalized by the protein size, N. (**b**) Total Lennard Jones potential energy as a function of $T_{m}$ for each protein of the $T_{m}$ dataset. The Pearson correlation is reported in the legend. (**c**) Same as in (**a**) but for the complexes of the $B_{a}$ dataset. (**d**) Same as in (**b**) but for the complexes of the $B_{a}$ dataset. (**e**) Probability density distributions of Lennard-Jones potential energy for the $T_{m}$ dataset (‘Intra’, green line) and between each couple of proteins of the $B_{a}$ dataset (‘Inter’, yellow line). Energies are considered only between couples of residues whose minimum distance is lower than 4 A, while energies regarding interactions between two close Cys residues have not been considered (see “Methods” for details). (**f**) Probability density distributions of Coulombic interaction energies for the protein of the $T_{m}$ dataset stratified from lower (blue) to higher (red) average $T_{m}$. Each distribution is built using a group of proteins whose melting temperatures lie in the same range; the average $T_{m}$ value of each group is reported in the legend. The inset shows the probability of finding a strong favorable/unfavorable interaction as a function of the average melting temperature of each subset. (**g**) Same as in (**f**) but for the complexes of the $B_{a}$ dataset.

Next, we compared the measured energies with experimental data of melting temperature and binding affinity for the $T_{m}$ and $B_{a}$ datasets, respectively. In particular, Fig. 1a and b show the total Coulombic and Lennard-Jones energies measured in each protein as a function of its experimental $T_{m}$. As one can see, a negative (respectively positive) linear correlation is present between the experimental thermal stability and the Coulomb (resp. Lennard-Jones) energy of each protein. More specifically, the Pearson’s correlation values are − 0.32 (with a *p* value of 0.03) and 0.30 (with a *p* value of 0.04) for the two cases.

Figure 1c and d, instead, report the total intermolecular Coulomb and Lennard-Jones interactions as a function of the experimental binding affinity, $B_{a}$ (defined as the $\log _{10}$ of the $K_{d}$).

Even in this case, linear correlations are studied between the two kinds of potential energies. In fact, a weak anti-correlation exists between the Coulombic intermolecular interactions and the experimental binding affinity values (Pearson correlation of − 0.08, with a *p* value of 0.056); while a positive Pearson correlation of 0.41 (*p* value $<\,10^{-6}$) is found between the van der Waals intramolecular interactions and the $B_{a}$ values of each protein complex. We note that although the signs of the correlations are the same, intra- and intermolecular interactions behave in the opposite manner. Indeed, lower values of $B_{a}$ correspond to higher binding affinity while higher values of melting temperature indicate a more stable complex. We extensively elaborate on this aspect in the “Discussion”.

Interestingly, results on the thermal stability hold also considering couples of homologous proteins coming from thermophilic/mesophilic organisms (see Supplementary materials for details).

Along the line of Miotto et al.^9^, we proceeded to examine the organization of the measured interactions, starting from an analysis of the probability distributions of finding a certain interaction energy between two residues. In Fig. 1e, we show the probability distribution of both LJ intramolecular (green line) and intermolecular (yellow line) interactions. The two curves are characterized by similar trends, where most of the area under the curve corresponds to negative (favorable) energy values. Therefore, both types of interaction show how the side chains optimize their spatial rearrangement to minimize the energetic contribution. However, there is a higher probability of observing strong favorable intramolecular interactions with respect to intermolecular ones. Repeating the same analysis on the Coulombic energies, we found that the distributions for the thermal stability and affinity datasets display the same overall shape. In particular, it is possible to identify four ranges of energies that correspond to four peaks of probability density, i.e. very strong favorable region ($< -100$ kcal/mol), a strong favorable energy region ($-100< E < -10$ kcal/mol), a strong unfavorable interaction region ($E > 10$ kcal/mol), and an intermediate region characterized by weaker (but much more probable) interactions (see insets on the right in Fig. 1f,g). The high favorable/unfavorable regions are centered around $\sim \pm \, 25$ kcal/mol.

To better investigate the relationship between thermal stability and Coulomb energy distribution of intramolecular interactions, as well as the relationship between binding affinity and Coulomb energy distribution of intermolecular interactions, we divided the $T_{m}$ dataset into four groups according to protein $T_{m}$. Then, the energy distribution was evaluated for each group. Similarly, the $B_{a}$ dataset was divided into five groups according to the binding affinity experimental values. Both temperature and affinity ranges were chosen in such a way to guarantee that each group was composed of a balanced number of proteins (or complexes), so as to allow consistent statistics when comparing the respective distributions.

Looking specifically at Fig. 1f, one can see that there is a marked dependence between thermal stability and the percentage of strong interactions. This is evident looking at the disposition of the density curves (Fig. 1f): the higher the thermal stability the higher the probability of finding strong interactions. On the contrary, less thermostable proteins possess a larger number of weak interactions.

Indeed, the probability of finding a high favorable/unfavorable interaction linearly depends on the protein melting temperature with a Pearson correlation coefficient of 0.97 and a *p* value of 0.03 (see inset in Fig. 1f).

Conversely, the probability density of Coulombic energies stratified by binding affinity ranges shows a trend opposite to that of the $T_{m}$ dataset. Indeed, as shown in Fig. 1g, the higher the binding affinity, the lower the maximum of the distribution peak in the range of strong interactions. As in the case of thermal stability, this behavior can be better observed in the inset, where the probability of finding high energy is shown as a function of the mean binding affinity of the complexes comprising each range. Also in this case, there is a Pearson correlation of 0.92 with a *p* value of 0.03. Again we note that more stable complexes have a lower $B_{a}$ value.

Finally, no appreciable trends were observed stratifying Lennard-Jones’ potential energy distributions according to thermal or affinity data.

### Energy organization at residue level

Next, we moved to consider a higher level of organization of the energies: instead of considering single interactions, we looked at how these interactions localize in each residue. With this aim, we computed a compact descriptor usually studied in network theory: the node strength. Thinking of residues in a protein as nodes in a network and of energies as the weights of the links connecting couples of nodes, we can define the node strength^9^ as $s_{i} = \sum _{j = 1}^{N_{aa}^{i}} E_{ij}$, where $N_{aa}^{i}$ is the number of residues found in interaction with residue *i* and $E_{ij}$ is the energy (i.e. the weights) of all the interactions that residue *i* shares with the nearby residue j.

**Figure 2:**
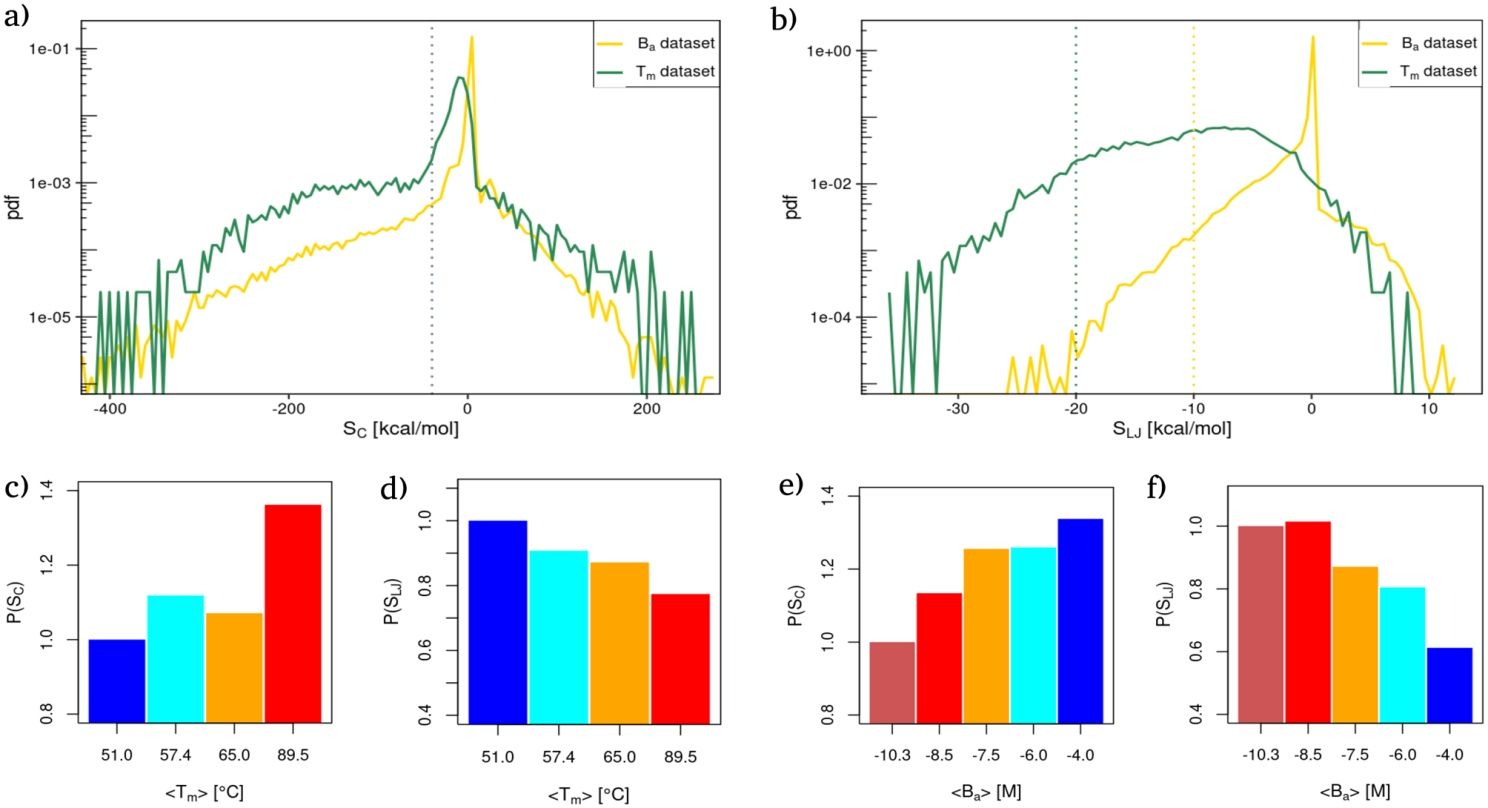
Energy reorganization at the residue level. (**a**) Probability distribution of the Strength values obtained using the Coulombic energies as network weights for the proteins of the $T_{m}$ dataset (green curve) and the complexes of the $B_{a}$ dataset (yellow curve). The grey dotted line delimits the left region of high favorable strengths. (**b**) Same as in (**a**) but considering Lennard Jones’s potential energy. (**c**) Relative probability of finding a residue with a high Coulombic strength value [dashed line in panel (**a**)] obtained stratifying the $T_{m}$ dataset in four intervals of increasing thermal stability (see “Methods” section). Relative probabilities are obtained dividing each probability by the probability of the group with the lowest mean thermal stability. (**d**) Same as in (**c**) but considering Lennard-Jones potential energies as network weights. (**e,f**) Same as in panel (**c**) and (**d**) but considering the complexes of the $B_{a}$ dataset, i.e. intermolecular interaction energies as network weights.

Similarly to what we have done for the interaction energies, in Fig. 2a (respectively Fig. 2b) we reported the probability density distributions of the strengths values for all the residues of the $T_{m}$ (green line) and $B_{a}$ (yellow line) datasets using Coulombic (resp. Lennard-Jones) potential energies as link weights.

Comparing the strength distributions with the ones in Fig. 1, one can see that the overall shapes of the distributions are similar, while they differ from the shapes of the interaction distributions, especially in the case of Coulombic potential energies. The latter in fact presented three distinct peaks in place of the single peak displayed by the strength distributions. Conversely, the shift between inter and intramolecular interactions are preserved for Lennard-Jones’ potential energies.

In all cases, the probability of finding residues with negative (i.e. favorable) strengths is higher than the one of finding residues with positive strength values, as one would expect in the case of stable folds and bindings. Interestingly, the distribution has tails that exhibit an exponential decay toward zero both for favorable and unfavorable strength values. This can be seen looking at the overall linear trend of the distribution tails in Fig. 2a,b, whose y-axis has been set to log-scale.

Even though the probability of finding high favorable strength values becomes exponentially smaller the more the strength is favorable, such probabilities show well-defined dependencies with respect to both thermal and binding stability.

In particular, Fig. 2c,d (respectively Fig. 2e,f) show the probability of finding a highly favorable Coulomb and Lennard-Jones strength as a function of the average melting temperature (resp. binding affinity) obtained stratifying the two datasets as done for the interaction energy distributions. High strengths are defined as all the strength values lower than the gray dotted lines in Fig. 2a,b), that mark the beginning of the favorable tails of the distributions.

It is interesting to note that the organization of the energies, measured by the network strength parameter, confirms the overall behavior observed when looking at total energies. More specifically, for protein structures with different thermal resistance, the greater the value of $T_{m}$, the greater the probability of finding high favorable strengths (see Fig. 2c). Contrary to the observed trend for Coulomb strengths, the greater the probability of finding negative Lennard-Jones strengths, the lower the thermal stability of the protein. Opposite trends are found in the case of binding, where the greater the probability of finding strong Coulomb strength, the lower the binding affinity of the complex (see Fig. 2e). Finally, Fig. 2f displays that the higher the probability of finding a residue with high favorable strength, the higher the complex binding affinity.

### Amino acid composition and hydropathy properties of the residues involved in intra- and intermolecular interactions

Given the different trends observed between Coulombic and Lennard-Jones interactions with respect to thermal stability and binding affinity, we investigated the amino acid composition of the proteins in the $T_{m}$ and $B_{a}$ datasets. In particular, we analyzed the frequency of occurrence of each amino acid and its hydrophobic/hydrophilic properties.

**Figure 3:**
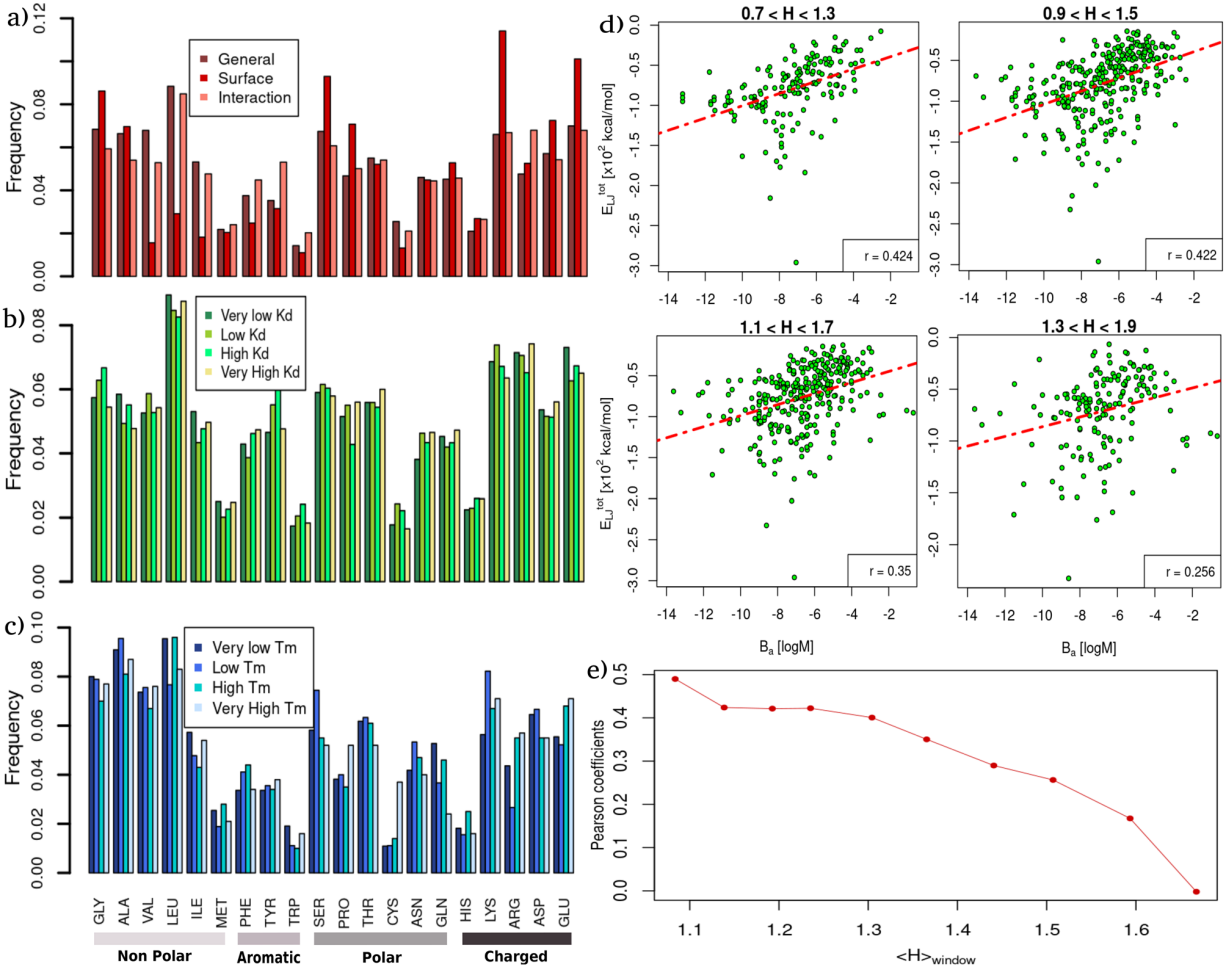
Comparing amino acid composition and hydropathy properties in proteins with different thermal stability and binding affinity. (**a**) Relative abundances of each of the twenty natural amino acids in the $T_{m}$ and $B_{a}$ datasets (see “Methods” section for details). For each kind of amino acid, the red bar corresponds to the abundance found in the solvent-exposed residues; the pink one refers to the residues found in interaction with the molecular partner, and dark red bars are computed considering all residues. (**b**) Relative abundances of each of the twenty natural amino acids found in the binding site regions of the $B_{a}$ dataset stratified by four groups of different binding affinities. Bar colors range from green to yellow as the $B_{a}$ of the considered complexes increases. (**c**) Relative abundances of each of the twenty natural amino acids found in the $T_{m}$ dataset, stratified by four groups according to the protein melting temperatures ($T_{m}$). Bar colors range from dark to light blue as the thermal stability of the considered proteins increases. (**d**) Total Lennard-Jones energies as a function of experimental binding affinity, $B_{a}$, considering only the complexes having mean hydropathy coefficient (H) within overlapping intervals of width 0.6. The Pearson correlation coefficients are reported for each plot. (**e**) Pearson coefficients against the mean hydropathy value of the window with respect to which the coefficient was calculated.

Figure 3a shows a general overview of the amino acid abundances using all the proteins in the two datasets. In dark red, the overall frequencies of residues occurrence in proteins are reported. In red, we recorded the values restricting to solvent-exposed residues (see "Methods" for the definition of superficial residue). In pink, we showed the frequencies of the amino acid observed to be in interaction in the $B_{a}$ dataset, where a residue is considered to be in contact if it has at least one atom closer than 4 Å to its molecular partner. This first analysis confirmed the well-known results that hydrophobic amino acids, such as Val or Leu, or Ile, are poorly present in the solvent-exposed surface of proteins. However, when a hydrophobic amino acid is present in the exposed regions it has a high probability of interacting with the corresponding molecular partner. On the contrary, charged amino acids, such as Lys or Glu, are typically more present in the superficial protein regions. This notwithstanding, the fraction of these actually participating in interaction is relatively small.

In addition, we studied the frequencies of amino acids found in protein binding sites, separated according to the binding affinity with partners (see Fig. 3b). Each bar represents a quartile of the distribution, meaning that the dark green to yellow bars regard the 25% of protein–protein complexes in our dataset with the lowest or highest $B_{a}$, respectively. No appreciable trends can be spotted between any amino acid abundance and the binding affinity classes. Lastly, in Fig. 3c, we investigated the frequencies of the amino acids in proteins characterized by different $T_{m}$. As in the previous plot, each bar represents a quartile of the $T_{m}$ distribution, from dark blue (very low $T_{m}$) to light blue (very high $T_{m}$). Interestingly, we found that a high presence of Cys is typical of proteins with very high $T_{m}$ since such a residue is responsible for the formation of stabilizing disulfide bridges. Moreover, the presence of a high number of charged residues, such as Arg or Glu, seems to be associated with a higher $T_{m}$.

Since we did not observe any robust trend between the amino acid composition of binding regions and the recorded binding affinity of the complex, we refined the analysis by dividing the $B_{a}$ dataset according to the average hydrophilic/hydrophobic properties of the complexes’ binding regions.

In order to do this, we associate each amino acid with a hydropathy index (*H*) according to the scale provided by Di Rienzo et al.^39^ (similar results were obtained considering canonical scales such as Kyte-Doolittle^40^ or Hessa et al.^41^; data not shown). The hydropathy scale is defined on the basis of a statistical analysis of the water molecule orientation and disposition around each kind of amino acid residue during molecular dynamics simulations. Thus the resulting hydropathy index depends on the environment usually found around each type of amino acid and not on the sole amino acid’s chemical-physical properties.

Ultimately, this scale indicates the propensity of the residues to interact with water and it is such that the higher the index the more hydrophilic is the residue, with $H = 0.0$ being the lowest value of the scale and associated with a purely hydrophobic behavior.

Figure 3d shows the total Lennard-Jones potential energy as a function of the complex binding affinity selecting the complexes in the dataset according to four different ranges of the mean hydropathy of their interface residues. One can clearly see that the Pearson correlation between the two quantities decreases the more the considered complexes have—on average—hydrophilic binding regions (see also Fig. 3e). That is, the affinity of complexes whose interfaces are mostly composed of hydrophobic residues is better described by van der Waals’s favorable energy with respect to hydrophilic ones.

### Binding region shape complementarity versus binding affinity

**Figure 4:**
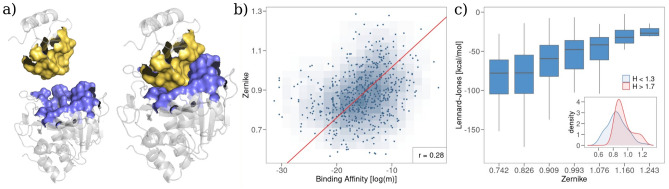
Comparison between shape complementarity and complex binding affinity. (**a**) Cartoon representation of the molecular surface of the binding region for a protein–protein complex (PDB id: 1TM1). (**b**) Minimum Zernike distance between the molecular surfaces of the binding regions as a function of the experimental binding affinity. (**c**) Box plot representation of the distributions of the total Lennard-Jones energy for different ranges of Zernike minimum distance. The inset reports the distributions of the Zernike values for the complexes whose binding region is mainly hydrophobic ($H < 1.3$) and mainly hydrophilic ($H > 1.7$).

Finally, leveraging on the results of the previous sections, we looked at the overall spatial organization of the interface residues of the complexes of the $B_{a}$ dataset. To do so, we evaluated the molecular surfaces of the two proteins forming the complex and measured the shape complementarity between the binding regions (see Fig. 4a). In particular, we use an algorithm, based on the formalism of Zernike polynomials in two dimensions^38^, which allows us to quantitatively characterize the morphological properties of small portions of molecular surface: the molecular patches are projected into a basis of orthogonal polynomials and the distance between the resulting vectors of coefficients representing the patches is evaluated. The shorter the distance between the Zernike descriptors, the greater the shape complementarity (see "Methods" for further details on the Zernike formalism). Operatively, we sampled a set of interaction patches from each binding region and we calculate the minimum distance between the vectors of the Zernike coefficients associated with corresponding patches.

Figure 4b shows the minimum Zernike distance as a function of complexes’ binding affinity. The two quantities share a linear relationship with a Pearson correlation of ~0.30 (*p* value $< 10^{-5}$), confirming that shape complementarity is a key factor for tuning binding affinity. It is worth noticing that shape complementarity is in turn linked to van der Waals interactions (see Fig. 4c). Finally, we note that shape complementarity is higher in complexes whose binding site is mainly composed of hydrophobic residues. This can be seen comparing the distributions of the Zernike minimum distances for complexes having low (hydrophobic behavior) or high (hydrophilic behavior) means Hydropathy index (see inset in Fig. 4c).

## Discussions

Several key structural features have been investigated and found to be related to the degree of protein thermal stability and protein–protein binding affinity. The former, in fact, increases as the protein packing decreases^42^, with the number of salt-bridges^8^, and thus with the presence of strong Coulombic interactions^9^. Binding affinity instead depends—among others—on the number of interactions taking place in the binding region^14^, the hydrophobic degree of the residues in interactions, and their distances^43^.

Intuitively, all the aforementioned descriptors are linked with the protein side-chain composition and organization, which is ultimately reflected in the interactions energies among residues and between residues and solvent. Here, we propose a comprehensive investigation and comparison between those aspects with respect to fold and binding stability. In particular, we collected two large datasets, one composed of monomeric proteins with known melting temperatures and another of dimeric complexes with recorded experimental binding affinity. Through those two refined collections of protein structures, we were able to perform a comparative analysis of the side-chain composition and organization, both in terms of amino acid abundances, chemical behavior, and interaction energy organization. In particular, the comparison between non-bonded interactions -the only kind of interaction present both in single-chain proteins and complexes—highlighted a clear difference in the role of Coulombic and van der Waals potential energies, which reflects also in the disposition of amino acids in the protein structures.

Indeed, the analysis of the interaction energies (see Fig. 1) clearly shows that both kinds of interactions are needed to ensure proper fold and binding. However, a marked difference is present between Coulombic and van der Waals interactions when one looks at the degree of stability of the fold/binding. In fact, boths present a correlation with the melting temperature (an indicator of the thermal stability of the fold) and the dissociation constant (a proxy for the stability of the protein complex), but the trends of those correlations are opposite. The higher the favorable Coulombic interaction energy the higher the thermal stability of the protein while to lower favorable van der Waals interactions correspond higher experimental $T_{m}$. Notably, this behavior of Lennard-Jones interaction is in agreement with previous observations about side chains packing: indeed, thermophilic proteins are less dense than their mesophilic counterparts^42^, i.e they possess a lower packing of the side chains which reflects in weaker LJ potential energies. The trend of Coulomb energy together with preferential localization of charged amino acid on the protein surface (see Fig. 3a) further suggests a cage-like effect of Coulomb interaction, which leads to an increased stabilization^9,44^. These conclusions are further strengthened by results on homologous proteins (see Supplementary materials). In particular, Supplementary Fig. 1 clearly shows that residues forming strong C interactions are preferentially located on the protein surface and that those residue are subject to evolutionary pressure.

In addition, we note that the Coulombic energies were computed without considering the solvent effect, thus this produced higher surface interactions than one would measure in more physiological conditions. However, since all our results stem from comparisons between protein groups we expect our result not to change qualitatively.

Notably, we found an opposite effect of Coulombic and van der Waals interactions when we looked at binding stability data. The presence of strong Coulombic interactions involving binding site residues seems to have a destabilizing effect on the complex stability. This is evident looking at the probability of finding high Coulombic energies, which strongly anti-correlates with the binding affinity (see Fig. 1g); it is also confirmed from strength data (Fig. 2e) and by the fact that even if charged residues are preferentially located on the protein surface, they are scarcely present in binding regions. Again, this is probably due to the fact that the function of these superficial charged residues is linked mainly with protein thermal stability and less with the strength of protein–protein binding^9^. It must be noted, however, that such charged residues could play a role in other phases of the binding process. For instance, the long-range nature of the electrostatic interactions is expected to be a driving force in the recognition step^45^. Moreover, a favorable electrostatic interaction may allow the two proteins to be in proximity of each other for a time long enough to permit the structural rearrangements necessary to bind^46^.

Complex with high favorable Lennard-Jones potential energies tend, instead, to be more stable than those having low van der Waals interactions^43^. A trend that is preserved also at the level of network strengths. At odds with what is observed for thermal stability, where the greater the probability of finding negative Lennard-Jones strengths, the lower the thermal stability of the protein.

These findings can be interpreted by looking at the dependence of the van der Waals interactions (modeled via the Lennard-Jones potential) on the distance between the interacting atoms. In fact, such potential is characterized by roughly three regimes: a strong repulsion at a low distance, an intermediate region of attractive force (around the characteristic radius of the interaction), and a third region where the interaction rapidly decays to zero. In this framework, our results confirm that thermostable proteins have an overall less dense packing since residues form weaker LJ interactions.

Moreover, since van der Waals interactions act on a short range, the interface protein atoms must be arranged in such a way that protein surfaces are compatible. The more the atoms are disposed around their respective Lennard-Jones characteristic radii the higher the van der Waals interaction and the higher the binding affinity. Notably, these relationships strengthen when the binding regions are composed of hydrophobic residues. The higher the hydrophobicity of the binding site residues, the more this behavior is evident (see Fig. 3d,e).

Altogether, our analyses identify van der Waals interactions as a key component to modulate complex stability. The relationship between shape complementarity, binding energy, and the van der Waals potential shown in Fig. 4 in turn confirms that shape complementarity is one of the key parameters to account for binding affinity.

As a corollary, we note that the found relationship may have important practical implications. In fact, to evaluate the shape complementarity between two interacting regions of each protein, we used the 2D Zernike formalism^38^. This procedure has an advantage over the direct computation of intermolecular interactions: the compactness of the description, as well as the low computational cost required, allows a direct comparison between all the possible interacting sub-regions belonging to the binding site. Moreover, the rotational invariance of the Zernike descriptors permits a super-position-free comparison between portions of the molecular surface of different proteins, i.e. there is no need to roto-translate the protein in space as needed for the evaluation of interaction energies. Those properties could allow for a direct application of the Zernike algorithm to the blind estimation of binding affinity on both experimental and/or docked protein complexes, at least for those where a lock-and-key mode of binding can be assumed.

Finally, one must consider that protein–protein interaction is a complex process that consists of various steps: distant proteins must recognize themselves in a crowded cellular space, sometimes large conformational changes must take place during the docking process, etc. Here, we only focused on the stability of the resulting complex, thus all our results do not necessarily hold for the other phases of the binding process. Moreover, it must be pointed out that the present study was carried out on sets of proteins having well characterized 3D structures, i.e. folding states, so again our results may not comprise the case of disordered proteins (even if evidences of ordering after interaction with partners have been reported for several disordered proteins^47,48^).

In conclusion, we proposed a comparative analysis of the composition and organization of the residues with respect to protein thermal or binding stability properties. We assembled two datasets with known experimental data of melting temperature and binding affinity and studied the influence of the chemical composition, energy/structural organization of residue/residue, and residue-solvent non-bonded interactions. In a nutshell, our work highlighted a prominent role of Coulomb interactions for thermal stability modulation, while the binding affinity between molecular complexes is mainly regulated by Lennard-Jones interactions. Finally, leveraging on the results of the energetic analyses, we compared the shape complementarity of the binding regions of the protein complexes, measured via the Zernike 2D method, and the experimentally measured binding affinities, finding a significant relationship between the two quantities. Overall, our results offer an overview of the molecular mechanisms that mediate protein/complex stability and may help guide novel predictive methods.

## Methods

### Datasets

To compare the residue organization and composition between protein fold stability and complexes with stable binding, we collected two datasets:

- The Thermostability ($T_{m}$) dataset, which consists of 49 monomeric structures with known melting temperature, $T_{m}$ (measured in Celsius degrees). To see how the different descriptors we defined (see next sections) on the $T_{m}$ dataset proteins, we grouped the structures according to their experimental melting temperature values. In particular, we defined four ranges, i.e very low thermal stability ($T_{m} < 55$), low thermal stability ($T_{m} \in [55,60]$), high thermal stability ($T_{m} \in [60,70]$), and very high thermal stability ($T_{m} > 70$).
- The Affinity ($B_{a}$) dataset consists of 567 complexes of known dissociation constant (measured in Molar units). Namely, the dissociation constant $B_{a}$, the inhibition constant $K_{i}$, and the half-maximal inhibitory concentration $IC_{50}$ were reported. To study how the investigated properties of the Affinity dataset’s complexes scale with complex stability, we grouped the structures according to their experimental binding affinity values. In particular, we defined five ranges, i.e very high binding affinity ($log_{10}(K_{d}) < -9$), high binding affinity ($log_{10}(K_{d}) \in [-9,-8]$), medium binding affinity ($log_{10}(K_{d}) \in [-8,-7]$), low binding affinity ($log{10}(K_{d}) \in [-7,-5]$), and very low binding affinity ($log{10}(K_{d}) > -5$).

The above ranges were chosen such that each contained a comparable number of items, in order to achieve consistent results.

The $T_{m}$ dataset was extracted from the most recent version of the proTherm database^49^. Filtering the database entries for crystallographic resolution (better than 3.0), pH range ($6.5< pH < 7.5$), known experimental $T_{m}$, absence of ions or/and ligands, absence of mutations and/or missing residues, and requiring that the structure is a monomer, we got 49 protein structures (11 novel monomer with respect to the dataset proposed in Miotto et al.^9^, where similar filters were used on a previous version of the ProTherm database) . In particular, 33 proteins have a $T_{m}$ lower than 70 $^{\circ }$C (i.e they are mesostable) and 16 have a higher one (i.e. they are considered thermostable). The ratio of found thermo/mesostable proteins is one over three, in accordance with what one would expect from literature knowledge.

The $B_{a}$ dataset was assembled starting from the data proposed by Dias et al.^34^, which consists in 622 protein complexes with known binding affinity. All non-dimeric complexes of the $B_{a}$ dataset were removed where no clear information was present on which couple of monomers the binding affinity refers to. From all proposed PDB structures, all heteroatoms were trimmed off. All PDB files were submitted to PDBfixer, which replaced non-standard residues and filled missing residues. After that, the datasets were subjected to renumbering of the sequence residues in order to remove all the inserts. Consequently, the complexes were minimized using GROMACS and the CHARMM force field^50,51^ in vacuum in order to remove structural alterations (like crystallographic clashes, etc.). Minimization was carried out using the steepest descent algorithm arresting the simulation when the maximum force was less than 1 kJ/mol. As for the $B_{a}$ dataset, structures having missing residues within 15 Å from the binding site were discarded. Distances were computed between the centroid of the missing residues and that of the binding site. The binding site was detected considering all the atoms placed at a distance lower than 4 Å from the atoms of the other chain, whereas a gap was represented as the centroid of the $\alpha$-carbons belonging to the two residues at the edges of that gap.

After these procedures, the $T_{m}$ dataset accounts for 49 protein structures while the $B_{a}$ dataset consists of 567 complexes. See Table I and Table II of the Supplementary materials for the PDB codes of the selected protein structures.

### Interaction energy calculation

Intra and intermolecular interaction energies were computed using the parameters obtained from the CHARMM force field. In particular, given two atoms $a_{l}$ and $a_{m}$ holding partial charges $q_{l}$ and $q_{m}$, the Coulombic interaction between them can be computed as:

1 $$\begin{aligned} \small E_{lm}^{C}= \frac{1}{4\pi \varepsilon _{0}}\frac{q_{l}q_{m}}{r_{lm}} \end{aligned}$$

where $r_{lm}$ is the distance between the two atoms, and $\varepsilon _{0}$ is the vacuum permittivity. Van der Waals interactions can instead be calculated as a 12-6 Lennard-Jones potential:

2 $$\begin{aligned} \small E_{lm}^{LJ} = \sqrt{\varepsilon _{l} \varepsilon _{m}}\left[ \left( \frac{R_{min}^{l} + R_{min}^{m}}{r_{lm}}\right) ^{12} - 2\left( \frac{R_{min}^{l} + R_{min}^{m}}{r_{lm}}\right) ^{6}\right] \end{aligned}$$

where $\varepsilon _{l}$ and $\varepsilon _{m}$ are the depths of the potential wells of $a_{l}$ and $a_{m}$ respectively, $R_{min}^{l}$ and $R_{min}^{m}$ are the distances at which the potentials reach their minima.

The total interaction energy between each couple of residues is defined as:

3 $$\begin{aligned} E_{AA_{ij}}^{X}= \sum _{l = 1}^{N_{atom}^{i} }\sum _{m = 1}^{N_{atom}^{j}} E_{lm}^{X} \end{aligned}$$

where $E_{AA_{ij}}^{X}$ is the energy between two amino acids *i* and *j*, obtained as the sum of the interactions between each atom of the two residues ($N_{atom}^{i}$, $N_{atom}^{j}$); *X* stands for the kind of interaction considered, either Coulombic ($X = C$) or Lennard-Jones ($X = LJ$).

As for the distance between a pair of residues, this was assessed by selecting the minimum distance between the atoms composing them.

### Hydropathy assessments

To assess the hydropathy of the interfaces, all the residues belonging to such regions were selected. Such selection was carried out considering all those residues on one protein partner having at least one atom at a distance lower than 4 Å from the atoms of the other. After that, the hydropathy index provided by the scale of Di Rienzo et al.^39^ was associated with the selected interface residues. The hydropathy of the interface was computed as the mean of the collected indexes. This process was performed on both the interfaces of a complex.

The selection of the complexes with respect to hydropathy for the energetic study was executed considering overlapping hydropathy windows of width 0.6 in the case of the scale of Di Rienzo et al. shown in the “Results” and “Discussions” sections. For the analysis, we considered the complexes having hydropathy index of both the interfaces within the window. The window approach was chosen in order to have a reasonable number of items in each window so as to obtain reliable statistical analyses.

### Computation of molecular surfaces and patch definition

The solvent-accessible surface for all proteins structure, given their X-ray structure in PDB format^52^, were computed through DMS^53^, using a density of 5 points per $A^{2}$ and a water probe radius of 1.4 Å . The unit normals vector, for each point of the surface, was calculated using the flag $-n$.

A residue is considered superficial if it has a Relative Solvent Accessibility higher than 0.25, when the Solvent Accessibility is calculated with DMS and the maximum Solvent Accessible Surface Area, for each amino acid, is taken from^54^.

The resulting molecular surface consists of a set of points in a three-dimensional Cartesian space (i.e. it is a discretization of the continuous molecular surface). Given a region of interest on this surface, we define a surface patch, $\Sigma$, as the points of the surface contained in the region of interest. In principle, the shape of the region of interest can be arbitrary, in this work we chose to use a spherical region of radius $R_{s} = 8 A$, centered on one point of the surface. The chosen $R_{s}$ has been found to yield optimum retrieval of the real binding region with respect to random ones in terms of shape complementarity^38^. Once the patch is selected, we compute the average of the external normal vectors and reorient $\Sigma$ in such a way that the average normal vector is aligned with the z-axis. Then, given a point *C* on the z-axis we define the angle $\theta$ as the largest angle between the perpendicular axis and a secant connecting *C* to any point of the surface $\Sigma$. *C* is then set so that $\theta =45^{\circ }$. Let us call *r* the distance between *C* and a surface point. We then build a square grid, and we associate each pixel with the mean *r* of the points it contains. In this way, a 2D function of the patch is obtained, that can be expanded on the basis of the Zernike polynomials: taking the norm of the coefficients of this expansion gives a set of Zernike descriptors, invariant under rotations in space. In the next section, we provide a brief summary of the main features of the Zernike basis. For more details see Refs.^38,55^.

### 2D Zernike polynomials and invariants

Given a function $f(r,\phi )$ expressed in polar coordinates and defined inside a unitary circle ($r < 1$), it is possible to represent the function in the Zernike basis as

4 $$\begin{aligned} f(r,\phi ) = \sum _{n=0}^{\infty } \sum _{m=0}^{m=n} c_{nm} Z_{nm} \end{aligned}$$

with

5 $$\begin{aligned} c_{nm} = \frac{(n+1)}{\pi } \langle Z_{nm}|f \rangle = \frac{(n+1)}{\pi } \int _{0}^{1} dr r\int _{0}^{2\pi } d\phi Z_{nm}^{*}(r,\phi ) f(r,\phi ). \end{aligned}$$

being the expansion coefficients. Zernike polynomials are complex functions, therefore they hold a radial and an angular part,

6 $$\begin{aligned} Z_{nm} = R_{nm}(r) e^{im\phi }. \end{aligned}$$

where the radial part for a certain couple of indexes, *n* and *m*, is given by

7 $$\begin{aligned} R_{nm}(r) = \sum _{k= 0}^{\frac{n-m}{2}} \frac{(-1)^{k} (n-k)!}{k!\left( \frac{n+m}{2} - k\right) !\left( \frac{n-m}{2}-k\right) !} r^{n-2k} \end{aligned}$$

In general, for each couple of polynomials, it can be shown that

8 $$\begin{aligned} \langle Z_{nm}|Z_{n'm'} \rangle = \frac{\pi }{(n+1)}\delta _{nn'}\delta _{mm'} \end{aligned}$$

which ensures that the polynomials can form a basis. Knowing the set of complex coefficients, $\{ c_{nm} \}$, a univocal reconstruction of the original image (with a resolution that depends on the order of expansion, $N = max(n)$) is allowed. We found that with $N=20$, i.e 121 coefficients, a good visual reconstruction of the original image is achieved.

By taking the modulus of each coefficient ($z_{nm} = |c_{nm}|$), a set of descriptors can be obtained which have the remarkable feature of being invariant for rotations around the origin of the unitary circle.

The geometric similarity between the two patches can then be assessed by comparing the Zernike invariants of their associated 2D projections. In particular, the similarity between patch *i* and *j* is measured as the Euclidean distance between the invariant vectors, i.e.

9 $$\begin{aligned} d_{ij} = \sqrt{\sum _{k=1}^{M=121} \left( z_{i}^{k} - z_j^{k}\right) ^{2}} \end{aligned} .$$

### Evaluation of local complementarity

When comparing patches, their relative orientation before the projection on the unitary circle must be taken into account. Intuitively, if we search for similar regions we must compare patches that have the same orientation once projected in the 2D plane, i.e. the solvent-exposed part of the surface must be oriented in the same direction for both patches, for example as the positive z-axis. On the contrary, if the complementarity between two patches is to be assessed, we must orient the patches contrariwise, i.e. one patch with the solvent-exposed part toward the positive z-axis (‘up’) and the other toward the negative z-axis (‘down’).

To evaluate the local complementarity of the Affinity dataset’s complexes, we first compute the molecular surfaces associated with each of the two protein structures forming the complex. Then, we identify the ‘template’ protein as the biggest of the two (in terms of the number of residues) while we refer to the smallest one as ‘ligand’. Next, we define the binding site of the template protein as the set of surface points whose minimum distance from any point of the ligand surface is lower than 3 Å. After that, for each point of the template binding region, we (1) define a template patch and its corresponding ligand patch taking all surface points that fall within a sphere of radius 8 Å centered in the considered template point. (2) we project the obtained patches in the 2D plane as described in the previous Section and expand them on the 2D Zernike basis. Finally, we compute the Euclidean distance between the 121 invariant coefficients associated with each patch.

Cycling over all the points of the identified binding regions, we end up with a set of Zernike scores (i.e. distances). Finally, for each complex, we compute the minimum of the found distances (see Fig. 4b).

## Supplementary Information

Supplementary Information.

## Data Availability

All codes and relevant data are within the Main Text, and at: https://github.com/matmi8/Zernike2D.
