# Supplementary material for: Spatial organization of hydrophobic and charged residues affects protein thermal stability and binding affinity
Source: Sci Rep. 2022 Jul 15;12:12087. doi: 10.1038/s41598-022-16338-5 (PMC9287411; doi:10.1038/s41598-022-16338-5)
Supplement: Supplementary file 1 — Supplementary Information. [file 41598_2022_16338_MOESM1_ESM.pdf]

Supplementary Materials -Spatial organization of hydrophobic and charged residues affects protein thermal stability and binding affinity

Fausta Desantis[1,3,+], Mattia Miotto[1,+,*], Lorenzo Di Rienzo[1], Edoardo Milanetti[2,1], and Giancarlo Ruocco[1,2]

[1]Istituto Italiano di Tecnologia (IIT), Center for Life Nano & Neuro Science, Viale Regina Elena 291, Roma, I00161, Italy
[2]Sapienza University of Rome, Department of Physics, Piazzale Aldo Moro, 5, Rome, I00185, Italy
[3]The Open University Affiliated Research Centre at Istituto Italiano di Tecnologia, Via Morego, 30, Genova,I16163, Italy
[*]mattia.miotto@roma1.infn.it
[+]these authors contributed equally to this work

Comparison between homologous pair

To further inquire on the role of non bonded interactions in protein thermal stability, we considered a specific case of study among the thermophilic monomers from the T_m dataset. This was chosen such that a mesophilic homologue counterpart was available (see the dataset proposed in (1)) in order to make comparison between the two cases. We consider 3-Isopropylmalate dehydrogenase from Thermus thermophilus (PDB id: 2y3z) and 3-Isopropylmalate dehydrogenase from the mesophilic Escherichia Coli (PDB id: 1cm7). For both proteins, we used the same procedure described in the Main Text for fixing, energy minimization and non-bondend interaction energy evaluation. Computing the total Coulombic and Lennard Jones energies, (see Fig. 1a and b), we found that Coulombic interactions take more negative values in the protein coming from the thermophilic organism with respect to the one belonging to the mesophilic counterpart. Lennard-Jones interactions behaves oppositely, in accordance with the general results found for different protein structures discussed in the Main Text. Indeed, lower Lennard Jones potential energies point to a less dense packing of the protein side chains, behavior already observed in previous studies on homologous pairs (2).

Interstingly, mapping the ten strongest Coulombic interactions on the protein structure, we found that the residues (depicted in red stick in Figure 1c) involved in these interactions are preferentially located on the protein surface. Taken together, these results further confirm the view that the stabilizing effect of Coulombic interactions is obtained by a cage-like disposition of strong C interaction on the protein surface. Moreover, the fact that the general finding we observed considering datasets of different protein structures remain valid also when comparing couples of homologous protein clearly shows the effect of the evolution process. In fact, those protein pairs share very similar fold, amino acid composition and biological function but are able to withstand different temperatures.

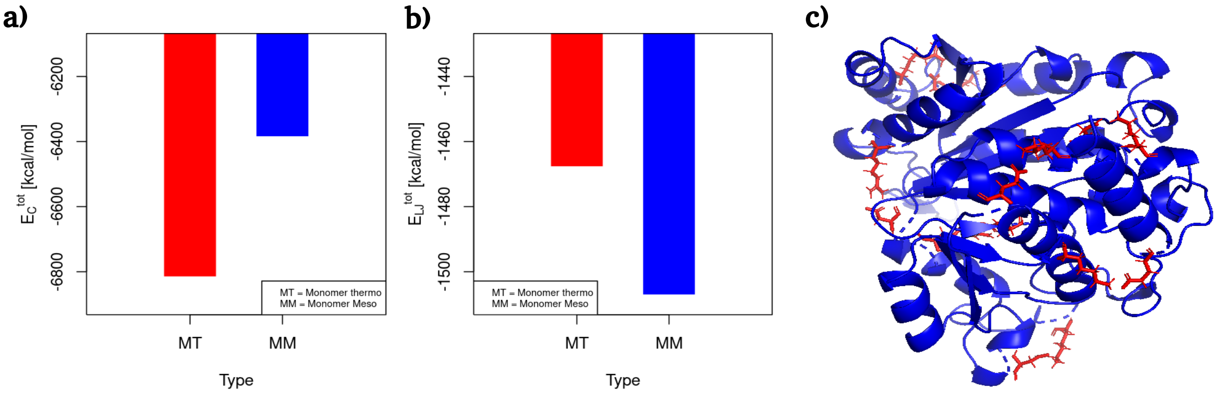

Figure 1. **Comparison between a couple of homologous proteins. a)** Total Coulomb interactions for a protein belonging to a thermophilic organism (PDB id: 2y3z) and its mesophilic homologue (PDB id: 1cm7) in red and blue respectively; **b)** same as in a) but with Lennard-Jones interactions; **c)** Three-dimensional ribbon-like rendering of 3-Isopropylmalate dehydrogenase from Thermus thermophilus (PDB id: 2y3z), with highlighted in red licorice sticks the residues that present the ten strongest Coulombic interactions.

Table 1. Affinity dataset with, in the order, PDB IDs, length of the chains composing the dimers (N_A,N_B), exprimental binding affinity (B_a).

PDB	N_A	N_B	B_a	PDB	N_A	N_B	B_a
1a22	180	192	3.4e-10	1g5j	175	25	6e-10
1abt	74	6	1.4e-06	1g6v	256	126	7.2e-08
1an1	223	40	9e-10	1g9i	223	22	1.2e-07
1atn	372	258	4.5e-10	1gag	303	13	7.5e-07
1avp	204	11	1.2e-08	1gbq	57	10	0.0035
1avx	223	172	6e-14	1gl0	241	32	8e-10
1ay7	96	89	1e-06	1gla	161	489	1.8e-07
1aze	56	10	6e-05	1grn	191	197	3.88e-07
1azg	14	58	1.6e-05	1gua	167	76	4e-08
1bjr	279	10	2.1e-08	1guw	73	18	1.9e-06
1buh	287	70	7.7e-08	1h0t	58	58	6e-06
1bvn	496	71	9e-12	1h1v	368	327	2.3e-08
1bxl	181	16	3.4e-07	1h3h	60	11	2.2e-07
1c9p	223	50	1e-09	1h6e	219	10	7e-07
1cka	57	9	1.9e-06	1haa	74	13	2e-09
1ckb	57	8	5.2e-06	1he8	749	166	2.5e-06
1clv	471	32	1e-09	1i3z	103	10	1.31e-07
1cwb	165	11	1.3e-08	1i4e	293	243	1.2e-07
1cwc	165	11	1.6e-08	1i5h	17	50	5.3e-05
1cyn	178	11	2.1e-08	1i8h	13	39	2.9e-05
1d4t	104	11	6.5e-07	1idg	74	18	6.5e-08
1d6r	223	58	1.3e-10	1irs	112	11	6e-06
1ddm	135	11	1.7e-06	1j19	316	16	1.64e-08
1dfj	124	456	5.9e-14	1j2j	165	41	1.4e-06
1djs	206	135	4.1e-08	1j4p	151	13	1.02e-05
1dp5	329	31	9e-10	1j4q	151	13	3.6e-07
1dpj	329	29	3e-09	1j7d	140	149	2e-06
1dpu	69	16	1e-06	1j7v	150	205	3.5e-11
1e96	178	185	2.7e-06	1j7z	15	101	2e-08
1ees	178	46	2e-08	1j80	15	101	3e-07
1ej4	179	14	5e-08	1j81	15	101	8e-08
1eja	223	53	1e-09	1jbd	74	14	1.2e-07
1es0	182	190	8e-08	1jgn	98	22	3.5e-07
1f34	326	138	1e-10	1jh4	98	22	1.5e-06
1f3v	158	171	7.8e-06	1jiw	105	470	4e-12
1f47	17	144	2.16e-05	1jm4	11	118	1e-05
1fc2	43	206	2.25e-08	1jsp	20	121	5e-05
1ff1	95	6	1.2e-05	1jtd	262	273	2.72e-11
1fmo	338	20	2.3e-09	1k3n	151	13	1.02e-05
1g0v	329	29	1e-10	1k3q	151	13	3e-07
1g1e	16	89	2.9e-08	1ka7	107	12	6e-07

**PDB**	$\mathbf{N}_A$	$\mathbf{N}_B$	$\mathbf{B}_a$
1kac	185	124	1.48e-08
1kbh	47	59	3.4e-08
1kna	52	6	7e-06
1kne	52	6	2.5e-06
1ktz	82	106	2.9e-07
1kxp	349	438	1e-09
1l0a	192	18	2.39e-05
1l2z	62	11	0.000203
1l4d	249	112	1.966e-07
1l4z	248	125	1.966e-07
1l8c	95	51	7e-09
1lcj	104	11	1e-09
1ldt	46	223	1.8e-09
1lp1	55	54	2e-06
1lw6	281	63	2e-12
1lx5	104	94	1.2e-09
1lxh	71	18	6.5e-08
1lzw	91	146	3.3e-07
1m10	199	267	5.8e-09
1m1e	512	65	1e-09
1m5n	28	485	2.1e-09
1mah	533	61	2.5e-11
1mik	165	11	4.5e-07
1mv0	14	81	9.33e-05
1mxl	89	17	0.000154
1mzw	173	31	1.97e-06
1nlo	56	6	3.4e-06
1nlp	56	6	1.1e-05
1ntv	152	10	1.5e-06
1o9a	93	24	1e-06
1oc0	364	37	1e-09
1oj5	105	14	8e-07
1om2	95	11	2e-05
1op9	121	130	7e-10
1oph	375	223	5e-09
1opi	104	13	5e-08
1oqp	77	19	5e-08
1otr	49	76	0.000155
1ozs	73	20	3.1e-05
1p4u	145	8	5e-06
1p69	185	124	7.6e-08
1p6a	185	124	3.5e-08
1p9d	32	78	1.29e-05
1pd7	85	24	3e-07
1pdq	51	9	5e-06
1pjm	20	427	1.8e-07

**PDB**	$\mathbf{N}_A$	$\mathbf{N}_B$	$\mathbf{B}_a$
1pjn	21	426	2.2e-08
1pmx	70	16	7.2e-06
1ppe	223	29	3e-12
1q0w	24	76	0.000277
1q5w	31	76	0.000126
1q68	38	29	4e-07
1q69	19	29	9e-07
1qng	170	11	1.3e-08
1qwe	56	12	1.2e-06
1qwf	56	12	4.5e-07
1r0r	274	51	2.94e-11
1r8u	50	100	1.3e-08
1rgj	74	13	4.9e-09
1ri8	124	129	2.9e-09
1rjc	123	129	7.7e-11
1rkc	258	26	3.9e-08
1ry7	151	213	2.3e-07
1s3k	212	216	2.4e-07
1s5q	16	89	5.2e-06
1sb0	87	25	1.5e-05
1sfi	223	14	1e-10
1shc	195	12	5.3e-08
1shy	228	499	9e-08
1smf	223	9	1.2e-07
1sq0	198	265	3e-08
1syq	259	25	1.47e-08
1t01	255	24	3.9e-08
1t0p	174	86	2.5e-05
1t44	358	144	1e-06
1t5z	250	11	3.3e-05
1t63	250	14	1.5e-05
1t6b	676	170	4e-10
1t79	250	8	9.2e-07
1t7f	250	8	1.8e-06
1t7r	250	10	1.1e-06
1ta3	274	301	9e-09
1taw	223	54	2e-11
1tba	67	180	1e-09
1tdq	271	126	1.2e-08
1te1	274	190	3.4e-09
1tlh	89	68	3e-04
1tm1	281	64	3e-12
1tm3	281	63	1.9e-11
1tm4	281	63	1.3e-10
1tm5	281	63	1.7e-11
1tm7	281	64	3.3e-12

PDB	N_A	N_B	B_a	PDB	N_A	N_B	B_a
1to1	281	63	4.6e-10	1yrt	351	69	1e-09
1u0i	21	21	7e-08	1yru	351	69	1e-09
1u0s	86	118	2.3e-07	1ywi	28	6	0.000371
1u5s	71	66	0.003	1yx5	112	76	0.00035
1uel	95	48	3.4e-06	1yx6	112	76	7.3e-05
1ugh	223	82	1.2e-11	1zgu	139	76	9.8e-05
1uj0	58	9	2.7e-05	1zhi	195	125	2e-07
1ukh	321	10	4.2e-07	1zjd	237	57	1.28e-09
1upk	318	4	4e-07	1zli	306	74	1.3e-09
1us7	206	193	3.37e-06	1zsg	65	22	7.5e-06
1usu	246	132	3.3e-06	1zub	109	6	2.7e-07
1uti	57	16	2.4e-06	1zv5	119	129	1e-08
1vet	122	118	1.28e-08	1zvh	124	127	7e-08
1veu	118	124	1.28e-08	1zvy	125	129	1e-10
1vg0	481	182	5e-09	2a0t	151	10	1.5e-05
1vrk	148	20	6.8e-09	2a24	107	108	3e-05
1vwf	121	8	3.1e-07	2a3i	253	12	9e-07
1wa7	60	22	9.58e-06	2a78	170	207	6e-08
1wa8	99	95	1.1e-08	2a7u	22	105	1.2e-07
1wlp	25	138	6.4e-07	2a9k	170	207	6e-08
1wpx	421	204	1.8e-09	2aq2	110	234	1.1e-08
1wq1	320	166	1.7e-05	2aq9	262	12	5e-08
1wqj	80	62	8.65e-07	2arp	105	148	4.3e-07
1wr1	76	58	1.48e-05	2asu	4	225	1e-09
1wrd	98	76	0.000409	2axi	92	10	1.4e-07
1x8s	98	9	8e-06	2b0z	294	108	1e-04
1xb7	215	9	6e-07	2b12	294	108	2e-05
1xdt	41	518	1e-08	2b42	364	184	1.07e-09
1xg2	317	151	5e-09	2b7c	437	90	4e-07
1xj7	256	9	3.9e-05	2b87	58	58	1e-07
1xr0	22	129	1e-05	2bba	185	14	7e-08
1xt9	208	76	2e-07	2btf	374	139	2.3e-06
1y1k	281	64	3e-10	2c0l	292	122	1.09e-07
1y2a	423	10	4.58e-08	2c1m	424	46	1.1e-09
1y33	281	64	1.7e-09	2c7m	58	73	6.4e-06
1y34	281	64	5.2e-10	2cpk	336	20	2.3e-09
1y3b	281	63	3.1e-10	2den	46	76	1.7e-05
1y3c	281	64	2.5e-11	2djy	42	20	4e-05
1y3d	281	64	5.6e-10	2dsp	91	57	3e-06
1y48	281	63	1.7e-09	2ez5	11	46	3e-06
1y4a	275	63	1.2e-09	2f31	228	20	1.02e-07
1y4d	276	63	1.2e-09	2f4m	295	61	6.5e-08
1y6k	145	205	1e-09	2fci	105	13	7e-08
1y6m	142	204	1.73e-07	2few	144	97	0.0037
1y6n	142	202	2.7e-08	2fju	177	696	5.3e-06
1y8n	374	97	1.17e-06	2flu	16	285	2e-08
1yc0	247	66	4e-09	2ftl	223	57	6e-14
1ycs	191	193	3e-08	2ftm	223	58	1.6e-11
1ydi	256	24	1.78e-09	2fts	419	13	9e-08

PDB	N_A	N_B	B_a	PDB	N_A	N_B	B_a
2fuh	146	76	3e-04	2kbr	80	18	2.54e-05
2fuu	62	15	2.7e-06	2kbw	160	31	3.2e-07
2fyl	81	82	2.8e-06	2kc8	95	33	2e-07
2g2u	265	165	1.25e-06	2kff	105	12	0.000245
2g2w	265	164	5.82e-07	2kfg	105	12	0.0012
2g6q	52	8	1.5e-06	2kfh	105	12	0.0024
2g81	223	55	1.36e-08	2khs	121	35	3.6e-07
2gng	341	20	6e-06	2kj4	87	32	6.5e-08
2gph	345	16	5e-06	2knb	76	62	1.1e-05
2hle	188	133	4e-08	2knh	103	18	7e-06
2hrk	177	121	9e-09	2koh	111	16	6e-06
2hsq	261	23	6.61e-09	2kpl	129	11	2.3e-07
2hth	73	129	0.000105	2krd	89	17	0.002
2hug	57	14	1.06e-06	2ks9	363	11	0.00902
2ixq	142	143	7e-07	2ksa	363	11	0.000871
2j12	182	120	2e-08	2ksb	363	11	0.000754
2jby	127	25	5e-08	2ksp	105	15	5.7e-05
2jgz	289	260	0.001	2ktf	76	32	2.3e-05
2jk9	202	8	1.5e-07	2kup	146	19	2e-07
2jnw	116	11	7.8e-07	2kvm	71	16	0.0001084
2jod	106	33	3.5e-07	2kwi	178	56	1.84e-07
2jq9	71	13	3.34e-05	2kwj	114	20	5e-07
2jqi	151	10	1e-05	2kwn	114	15	7.4e-06
2jqk	72	14	0.000402	2kwo	114	20	4.69e-05
2jql	141	10	3e-07	2kwu	36	76	5.1e-05
2jt4	71	76	4e-05	2kwv	36	76	9e-05
2jti	294	103	5.88e-07	2kxh	199	31	1.4e-05
2ju0	175	52	1e-07	2kxw	73	27	1e-08
2jy6	76	52	2e-05	2kzu	94	18	6e-05
2k00	92	15	2.4e-05	2l0f	76	45	0.000172
2k2r	129	10	0.0013	2l0i	136	9	1.5e-05
2k2s	136	55	5.3e-08	2l11	27	127	7e-09
2k2u	115	35	3.6e-07	2l6e	94	14	1e-06
2k3s	119	67	2.7e-06	2l9s	45	94	2.2e-06
2k3u	91	22	8.4e-09	2nm1	430	17	3.4e-08
2k3w	73	12	5.8e-06	2nqd	109	221	3.9e-11
2k42	72	36	3.5e-08	2o3b	241	135	3.2e-12
2k5b	210	129	4e-06	2o9v	67	10	0.000288
2k6d	62	76	0.000171	2oi3	86	12	2.3e-07
2k79	63	108	0.00067	2omt	461	104	1e-07
2k7a	63	108	0.00067	2omu	462	105	6e-10
2k7l	67	19	7e-07	2omw	461	105	1e-05
2k8b	76	80	0.0018	2omx	462	107	1.2e-07
2k8c	76	80	0.0018	2omy	460	104	2e-07
2k8f	90	39	2.7e-06	2omz	465	104	4e-07
2ka4	100	57	5.8e-08	2oob	44	72	5.7e-05
2ka6	92	45	5.2e-08	2ot3	253	157	1.8e-06

PDB	N_A	N_B	B_a	PDB	N_A	N_B	B_a
2oza	332	340	2.5e-09	2vog	145	22	2.1e-07
2p43	124	123	2.3e-08	2voh	155	24	1e-09
2p44	119	123	2e-08	2voi	150	24	1.1e-09
2p45	124	121	1.16e-07	2vsm	413	137	3.5e-08
2p47	123	121	1.16e-07	2vwf	56	14	8.7e-06
2p48	124	121	1.16e-07	2w85	57	12	9.17e-06
2p49	119	123	2.3e-08	2w9r	97	11	4.8e-06
2p8q	873	39	1.56e-08	2wel	304	145	6e-07
2pon	23	156	1.4e-06	2wfj	172	11	7e-08
2pr9	254	10	4.22e-08	2wfx	152	417	1.4e-08
2ptc	223	58	6e-14	2wg4	145	421	7.39e-08
2ptt	103	108	4e-06	2wh6	157	22	1.8e-08
2px9	217	158	8.74e-05	2wo2	178	136	1.08e-05
2qic	51	8	3.3e-06	2wo3	176	142	2.3e-06
2qna	744	30	8.3e-08	2wp3	97	99	7.94e-07
2qur	340	20	5e-07	2wpt	82	113	1e-07
2qxv	352	29	3.8e-07	2wwk	99	99	1.28e-06
2r02	697	11	8e-06	2wwx	169	200	7.9e-08
2r05	697	11	7e-06	2wy8	292	66	3.6e-07
2rmk	192	81	1.7e-07	2x1x	103	201	1.6e-08
2rms	71	61	1.34e-07	2xgy	129	165	3e-05
2rnr	62	108	1.5e-07	2xs8	697	9	2.45e-05
2rnw	118	15	0.001051	2xtt	35	223	2e-11
2rnx	118	13	0.000402	2y9m	166	117	2e-09
2rol	64	12	2.4e-05	2z58	318	66	1.6e-08
2roz	32	136	3.2e-07	3ajb	293	26	4.08e-08
2rr3	128	44	2.1e-06	3alz	417	109	5.2e-07
2sic	275	107	7.12e-11	3aon	188	101	3.2e-09
2sni	275	64	2e-12	3bh6	161	314	9.5e-08
2tgp	58	223	2.4e-06	3bn3	180	196	2e-05
2uuy	223	52	5.6e-09	3bs5	86	75	9.25e-08
2uyz	156	78	8.2e-08	3btr	15	427	5e-06
2v3b	381	53	5e-06	3bu6	297	14	1.3e-06
2v4z	316	122	1.25e-06	3bum	9	304	3.2e-07
2v52	360	30	1.9e-06	3bun	7	304	6.1e-07
2v8s	137	93	2.2e-05	3bzd	109	234	9.6e-05
2v9t	107	210	8.2e-09	3c3o	357	13	4.4e-05
2vay	146	21	7.9e-09	3c3q	357	18	4.8e-05
2vda	828	28	3e-06	3c3r	357	13	4.1e-05
2vdb	574	55	1.5e-10	3c4o	265	165	4.6e-09
2ver	143	110	1.31e-05	3c4p	265	165	4.4e-09
2vln	80	134	1.68e-12	3c59	103	27	5e-09
2vlo	90	132	8.92e-13	3c5t	104	25	6e-10
2vlp	82	134	5.43e-13	3cfs	383	15	1.1e-06
2vlq	84	134	2.28e-11	3ch5	193	37	0.00012

PDB	N_A	N_B	B_a	PDB	N_A	N_B	B_a
3cqc	247	186	4e-09	3jzs	83	12	8e-09
3cqg	225	184	4e-09	3k1r	192	74	1e-09
3cs8	269	12	1.7e-06	3k26	360	2	4e-04
3d7t	249	265	7e-05	3k8p	295	594	1e-08
3ddc	166	133	7.7e-07	3kj0	157	24	2e-09
3di3	118	193	2.1e-08	3kj1	152	22	2e-09
3doe	189	114	2e-08	3kj2	151	22	2e-09
3dow	119	7	1.15e-05	3knb	98	96	9.4e-07
3ds0	82	12	1.49e-05	3kuc	167	76	4.42e-07
3ds1	81	11	2e-06	3kud	165	76	1.7e-06
3e1z	109	212	3.6e-11	3kv4	432	14	1e-06
3eba	121	130	2.36e-09	3kw5	223	76	3.85e-07
3eg6	304	9	1.7e-06	3l3x	250	12	7.1e-07
3emh	300	6	1.4e-07	3l3z	249	12	1.36e-05
3eqs	84	11	3.3e-09	3l6x	434	18	4e-05
3eu7	313	14	6.6e-07	3l9j	130	149	3.4e-10
3f1p	114	111	0.00012	3lbx	140	182	4e-07
3fhc	381	224	2.16e-08	3lms	310	74	1.2e-09
3fii	403	27	1.9e-09	3m18	245	127	1.8e-10
3fju	307	65	1.6e-09	3mca	377	341	3.9e-07
3fp6	223	58	1e-13	3me2	156	166	6.8e-11
3fpu	100	66	1.2e-10	3mj7	229	189	5e-06
3g7l	55	7	1.9e-07	3mzg	186	206	0.00497
3gb8	958	267	6.5e-06	3mzw	569	53	2.2e-11
3gbq	57	10	3.5e-06	3n06	186	206	0.0031
3gj3	202	27	4.9e-05	3n0p	186	206	0.0051
3gj6	203	26	6.5e-06	3n4i	265	165	1.1e-09
3gni	335	304	1.2e-08	3ncb	186	206	0.093
3gqi	307	226	3.3e-08	3ncc	186	206	0.212
3gty	149	412	4.7e-10	3nvn	383	474	9.4e-09
3gxu	171	138	2.03e-07	3o34	177	12	8.8e-06
3h1z	233	15	2.2e-05	3oap	214	11	5.5e-07
3h8k	164	28	2.1e-08	3ob1	10	296	3.83e-06
3hct	104	148	1.48e-06	3ohm	318	759	2e-07
3hqh	134	9	6.36e-05	3oiq	183	30	3.8e-06
3hs8	238	12	5e-06	3oky	609	540	1.3e-06
3hs9	233	11	4.9e-07	3ol2	614	496	5.5e-06
3i5r	81	9	4e-05	3olm	412	74	9.06e-05
3idb	343	149	1.13e-08	3ona	158	66	6.8e-07
3idc	343	159	6e-10	3oux	503	47	3.5e-08
3iiw	359	5	3.64e-05	3p71	303	315	3.8e-07
3iiy	364	8	1.6e-05	3p92	224	58	5.9e-09
3ij0	359	5	1.32e-05	3p95	224	58	1.5e-06
3ij1	359	7	2e-05	3qc8	170	80	1.12e-05
3iol	100	26	5e-07	3qq8	177	80	1.5e-06
3iqq	89	9	2.9e-06	3qsk	119	123	9.1e-08
3ixe	171	70	2.3e-06	3rgf	321	266	7.05e-09
3jpx	359	6	0.000235	3t04	98	103	5.2e-08
3jza	168	193	3e-12	3tac	324	292	5.5e-07
3jzo	87	12	3e-09	3tnf	170	379	1e-11
3jzp	87	12	2.5e-07	3uyo	114	94	6.8e-06
3jzr	97	12	3.6e-08	3zyi	395	303	7.3e-09

Table 2. Thermostability dataset with, in the order, PDB IDs, length of the monomeric chain (N_{res}), organism to which it belongs, metling temperature (T_m), type of organism with M and T standing for mesophilic or thermophilic organism (definition according to (3) and (4))

PDB	N_{res}	Organism	T_m	Type
1ako	268	Escherichia coli K-12	42.6	M
1b8e	152	Bos Taurus	78	M
1bd8	156	Homo Sapiens	51.9	M
1bk7	190	Momordica Charantia	64.4	M
1bpi	58	Bos Taurus	104	M
1btl	263	Escherichia coli	56.4	M
1c5g	379	Homo Sapiens	67.5	M
1cec	331	Actevibrio Thermocellus	70.4	T
1cm2	85	Escherichia coli	63.4	M
1csp	67	Bacillus Subtilis	52.8	M
1div	149	Geobacillus Stearothermophilus	77.6	T
1ekg	119	Homo Sapiens	58.2	M
1ew4	106	Escherichia coli	53.8	M
1fsf	266	Escherichia coli	66.9	M
1h09	338	Streptococcus phage Cp-1	51.4	M
1h7m	99	Thermococcus celer	93.5	T
1hk0	173	Homo Sapiens	80	M
1ino	175	Escherichia coli	58	M
1j2v	102	Pyrococcus horikoshii OT3	148.5	T
1jyd	174	Homo Sapiens	68.9	M
1mjc	69	Escherichia coli	59	M
1msi	66	Zoarces americanus	46.6	M
1npk	150	Dictyostelium discoideum	62	M
1onc	104	Lithobates pipiens	88.5	M
1orc	60	Escherichia virus Lambda	57	M
1pii	452	Escherichia coli	51	M
1poh	85	Escherichia coli	63.4	M
1qhe	168	Nostoc sp. PCC 7119	56	M
1rop	56	Escherichia coli	68.7	M
1rtb	124	Bos Taurus	61.3	M
1sfp	111	Bos Taurus	78.6	M
1stn	136	Moesziomyces antarcticus	52.7	M
1tca	317	Moesziomyces antarcticus	57.7	M
1tpe	249	Trypanosoma brucei brucei	51.4	M
2cro	65	Phage 434	56	M
2dri	271	Escherichia coli	57.5	M
2lzm	164	Escherichia virus T4	62.2	M
2prd	174	Thermus thermophilus HB8	86	T
2sil	381	Salmonella enterica subsp. enterica serovar Typhimurium	57	M
2y3z	351	Thermus thermophilus HB8	87	T
3chy	128	Escherichia coli	57.8	M
3d2a	179	Bacillus subtilis	63.4	M
3enj	438	Sus scrofa	47.8	M
3kvd	242	Neisseria meningitidis	84.1	M
3n4y	98	Escherichia coli	100.2	M
3ssi	108	Streptomyces albogriseolus	82.2	M
4gcr	174	Bos Taurus	70.4	M
4lyz	129	Gallus Gallus	80	M
5pep	326	Sus scrofa	52	M

References

1. Miotto, M. *et al.* Simulated epidemics in 3d protein structures to detect functional properties. *J. Chem. Inf. Model.* (2019).

2. Amadei, A., Galdo, S. D. & D'Abramo, M. Density discriminates between thermophilic and mesophilic proteins. *J. Biomol. Struct. Dyn.* **36**, 3265–3273 (2017).

3. Willey, J., Sherwood, L. & Woolverton, C. *Prescott, Harley, and Klein's Microbiology*. Higher education (McGraw-Hill Higher Education, 2008).

4. Madigan, T., Martinko, J. & T.M., B. *Brock biology of microorganisms*. Higher education (Pearson, Prentice Hall, 2006).
